# Apparent Diffusion Coefficient (ADC) and Magnetic Resonance Imaging (MRI) Nomogram for Differentiating a Solitary Fibrous Tumor (World Health Organization Grade II) From an Angiomatous Meningioma

**DOI:** 10.7759/cureus.79470

**Published:** 2025-02-22

**Authors:** Yu Ying, Noorazrul Yahya, Hanani Abdul Manan

**Affiliations:** 1 Department of Interventional Radiology, University Kebangsaan Malaysia Medical Centre, Kuala Lumpur, MYS; 2 Department of Diagnostic Imaging and Radiotherapy, Centre for Diagnostic, Therapeutic and Investigative Sciences, Faculty of Health Sciences, National University of Malaysia, Kuala Lumpur, MYS; 3 Functional Image Processing Laboratory, Department of Radiology, National University of Malaysia, Kuala Lumpur, MYS

**Keywords:** diffusion-weighted imaging, magnetic resonance imaging, meningioma, nomogram, solitary fibrous tumor

## Abstract

Introduction: Accurate preoperative differentiation between intracranial solitary fibrous tumor (SFT, World Health Organization grade II) and angiomatous meningioma (AM) is crucial for surgical planning and prognosis prediction. While conventional magnetic resonance imaging (MRI) is widely used, distinguishing these tumors based on imaging alone remains challenging. This study aimed to evaluate clinical and MRI features to improve diagnostic accuracy between SFT and AM, focusing on the apparent diffusion coefficient (ADC) and conventional MRI parameters.

Methods: A retrospective analysis was conducted on 51 patients (23 with SFT and 28 with AM) confirmed by pathology. Clinical and MRI characteristics were assessed using t-tests and chi-square tests. Logistic regression analysis was performed to identify independent predictors, and receiver operating characteristic (ROC) curve analysis evaluated diagnostic performance. A nomogram integrating ADC values with conventional MRI features was developed and validated using calibration curves.

Results: Significant differences in tumor shape, cystic necrosis, T1-weighted imaging and T2-weighted imaging signal intensities, and ADC values were observed between SFT and AM (p < 0.05). Logistic regression analysis confirmed these factors as independent predictors, with ADC demonstrating the highest diagnostic performance at an optimal cutoff value of 1.08 × 10^-^³ mm²/second. The ROC analysis showed that combining ADC with conventional MRI features improved diagnostic accuracy. The calibration curve demonstrated strong agreement between nomogram predictions and actual outcomes.

Conclusion: Integrating ADC values with clinical and MRI features provides a reliable method for differentiating intracranial SFT from AM. This approach enhances diagnostic precision, aiding in optimized clinical decision-making and surgical planning.

## Introduction

SFT (World Health Organization, WHO, grade II) is a rare type of tumor originating from Zimmermann cells in the capillaries of the meningeal septum and accounts for approximately 1% of tumors of the central nervous system (CNS), which was formerly named hemangiopericytoma (HPC). HPC has been found to share inversions on the long arm of chromosome 12 (12q13) and a fusion of the NAB2 and STAT6 genes with the solitary fibrous tumor (SFT). In 2016, the WHO classified the CNS tumors, specifically HPC and SFT, into one unit called SFT/HPC [1]. However, in 2021, the WHO classification of CNS tumors SFT/HPC was renamed SFT [2].

SFT (WHO grade II) is difficult to differentiate clinically from angiomatous meningioma (AM). AM is a benign tumor classified as WHO grade I, and a good prognosis can be achieved with simple surgical resection. In contrast, SFT (WHO grade II) tends to exhibit aggressive behavior, with a high rate of recurrence and extracranial metastasis [3-5]. In addition, both SFT (WHO grade II) and AM are vascularized tumors, increasing the risk of intraoperative bleeding and causing patients to be more prone to shock or death [6]. Therefore, accurate differential diagnosis of these two tumors is especially important for planning appropriate surgical procedures, minimizing the risk of bleeding, and improving patient prognosis.

Magnetic resonance imaging (MRI) can provide the location, size, cystic necrosis, and invasion of surrounding tissues of tumors, providing abundant information for clinical diagnosis [7]. Advanced MRI techniques have significantly enhanced the accuracy of tumor characterization, aiding in differential diagnosis, treatment planning, and prognosis assessment. Diffusion-weighted imaging (DWI) measures water molecule movement. It helps distinguish high-grade from low-grade gliomas based on apparent diffusion coefficient (ADC) values. In contrast, diffusion tensor imaging provides insights into white matter tract integrity, assisting in preoperative planning by mapping critical fiber pathways. Perfusion-weighted imaging evaluates tumor vascularity through cerebral blood volume and cerebral blood flow (CBF), facilitating tumor grading and monitoring treatment response. Magnetic resonance spectroscopy analyzes tumor metabolism, distinguishing aggressive tumors by altered choline (Cho) and N-acetylaspartate levels. In contrast, arterial spin labeling perfusion MRI offers a noninvasive method for assessing CBF without contrast agents, which is particularly useful for patients with renal impairment. Functional MRI detects brain activity by measuring blood oxygenation changes, aiding surgical planning by identifying eloquent brain regions involved in motor and language functions. These advanced imaging modalities, when integrated, provide a comprehensive approach to tumor assessment, improving diagnostic accuracy, guiding therapeutic strategies, and enhancing patient outcomes [8]. Some researchers have studied the role of ADC measurements in the differential diagnosis of SFT (WHO grade II) and AM with this technique and obtained valuable results [9,10]. However, most studies focus on a single sequence study or differential diagnosis based on the difference in imaging features, and few studies combine imaging information with clinical features to construct differential diagnosis models. Therefore, this study aims to develop a combined clinical and MRI-based model to differentiate SFTs (WHO grade II) from atypical meningiomas (AM), thereby improving the accuracy of SFT (WHO grade II) diagnosis.

## Materials and methods

Study population

This study has been approved by the Institutional Review Board at the Hospital of Jining Medical University and adheres to ethical committee standards. The requirement for written informed consent was waived. From June 2019 to June 2023, 51 patients with SFT (WHO grade II) and AM, confirmed by pathological examination, were recruited. In total, there were 23 SFT (WHO grade II) patients, including 13 male and 10 female patients, with a mean age of 44.7 ± 15.6 years. There were 28 patients with AM, including 12 male and 16 female patients, with a mean age of 48.3 ± 12.6 years. The inclusion criteria were as follows: 1) the absence of surgical or other treatment before MRI examination and 2) no contraindication of MRI examination. The exclusion criteria were as follows: 1) allergic constitution, 2) recurring tumors or multiple lesions, 3) unconsciousness and communication disorder, and 4) low-quality or unclear MRIs.

Magnetic resonance imaging

All patients underwent MRI before clinical intervention on a 3.0-T MRI scanner (Discovery MR 750w, GE Healthcare, Milwaukee, WI) with a 32-channel head coil. Imaging parameters were as follows: T1-weighted imaging (T1WI; repetition time/echo time, TR/TE, 400/9 ms), T2-weighted imaging (T2WI; TR/TE, 4,660/110 ms), fluid-attenuated inversion recovery (TR/TE, 8,000/95 ms), matrix = 256 × 256, slice thickness = 5 mm, and slice gap = 2 mm. DWI was performed before contrast agent administration using single diffusion-weighted echo-planar imaging with the following parameters: TR/TE, 10,000/88 ms, slice thickness 5 mm, field of view 240 mm, matrix 130 × 128, and b-value 0 and 1,000 mm^2^/second. Then, gadolinium-enhanced T1WI scans were obtained after injection of a contrast agent (gadolinium-diethylenetriaminepentaacetic acid; 0.2 mL/kg body weight; Magnevist; Bayer Schering, Guangzhou, China). Table 1 summarizes the MRI parameters.

**Table 1:** Summary of the MRI parameters TR/TE: repetition time/echo time; T1WI: T1-weighted imaging; T2WI: T2-weighted imaging; FLAIR: fluid-attenuated inversion recovery; DWI: diffusion-weighted imaging; Gd-DTPA: gadolinium-diethylenetriaminepentaacetic acid; MRI: magnetic resonance imaging

Sequence	TR/TE (ms)	Matrix	Slice thickness (mm)	Slice gap (mm)	Field of view (mm)	b-value (mm²/second)	Contrast agent
T1WI	400/9	256 × 256	5	2	N/A	N/A	No
T2WI	4,660/110	256 × 256	5	2	N/A	N/A	No
FLAIR	8,000/95	256 × 256	5	2	N/A	N/A	No
DWI	10,000/88	130 × 128	5	N/A	240	0 and 1,000	No
Gadolinium-enhanced T1WI	N/A	N/A	N/A	N/A	N/A	N/A	Gd-DTPA (0.2 mL/kg)

Image analysis

The MR images were analyzed by Y.Y., N.Y., and H.M. with 7, 11, and 11 years of neuroradiology experience, respectively; the signal and the ADC values of SFT (WHO grade II) and AM were recorded. In addition, the following parameters were also recorded: tumor volume, edema index (EI), shape (lobulated, circular), peritumoral space, tumor signal homogeneity, enhanced homogeneity, cystic necrosis, hemorrhage, calcification, flowing void effect, attachment mode to the meninges, dural tail sign, and adjacent bone changes. Tumor volume was defined as V = length × width × width/2 [11]. The EI quantifies peritumoral edema relative to tumor size and is calculated as EI = (Total Edema Volume + Tumor Volume) / Tumor Volume, with higher values (>1) indicating significant edema. Tumor margins and interfaces with brain tissue are assessed for invasiveness; well-defined margins suggest noninfiltrative tumors like meningiomas, while poorly defined margins indicate aggressive tumors like gliomas. The peritumoral space helps distinguish invasive from noninvasive tumors, and dural attachment (e.g., broad-based in meningiomas) differentiates extra-axial from intra-axial tumors. The dural tail sign suggests meningiomas, while cystic/necrotic changes are common in high-grade gliomas, indicating aggressive behavior. These factors aid in tumor classification, surgical planning, and prognosis. EI was defined as the EI = (V_edem + V_tumor) / V_tumor [12], where V_edem indicates the volume of edema and V_tumor indicates the tumor volume. Attachment mode to the meninges was defined as broad and narrow. A broad base was described as an obtuse angle between the edge of the tumor and the meninges, while a narrow base was characterized by the acute angle between the two. According to a previous study [13], numerical scores of 1-5 were used to measure tumor signal intensity. Contrast enhancement in meningiomas was significant when it exceeded that of the cavernous sinus, moderate when it was similar to that of the cavernous sinus, and mild when it was less than that of the cavernous sinus.

**Figure 1:**
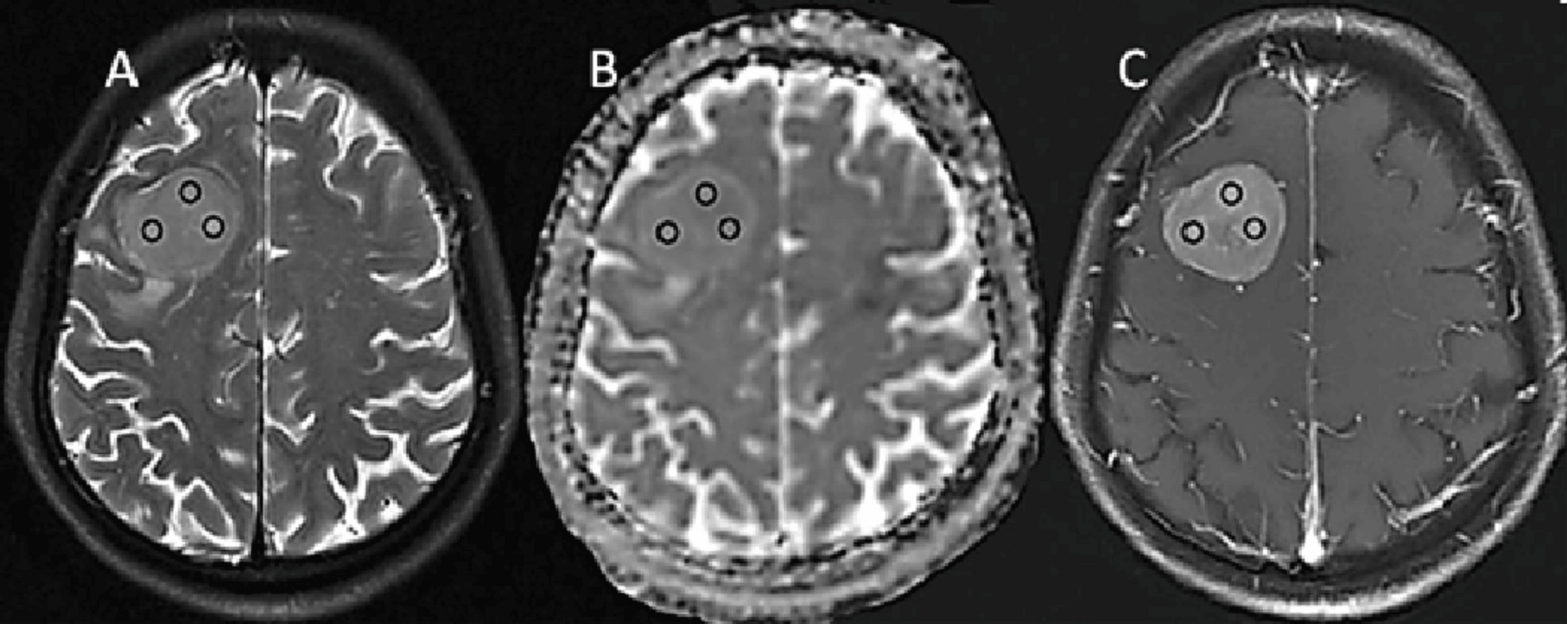
Example of ROI delineation. Three ROIs (A-C) were placed in the Gd-enhancing tumor part (black circle) based on co-recoded traditional images randomly ROI: region of interest

DWI images were transferred to the AW 4.6 Workstation, and an ADC map was acquired using vendor-provided software (Function tool ADC software; GE Healthcare, Chicago, IL). Simultaneously, three regions of interest (ROIs) were randomly distributed in the tumor core based on Gd-T1WI and T2WI coregistered with ADC images, avoiding areas of hemorrhage, cystic necrosis, calcification, and flowing void effect. Images were registered by aligning Gd-T1WI, T2WI, and ADC maps using coregistration techniques to correct spatial misalignment and ensure accurate overlay of anatomical structures. Three ROIs were randomly placed within the tumor core, carefully avoiding areas of hemorrhage, cystic necrosis, calcification, and flow void effects to ensure reliable measurements. This process enhances consistency in analyzing tumor characteristics across different MRI modalities. The ADC value of three ROIs was recorded for each patient, and then, the average value was determined as (ADC1 + ADC2 + ADC3) / 3 (Figure 1).

Statistical analysis

Statistical analyses were performed using a statistical analysis software package (Statistical Package for the Social Sciences 26.0, IBM Corp., Armonk, NY) and R version 4.0.0 (R Foundation for Statistical Computing, Vienna, Austria; http://www.r-project.org/). The objectivity and reliability of measuring materials between two observers were analyzed using the Cronbach alpha and the standardized Cronbach alpha. The Cronbach alpha and the standardized Cronbach alpha >0.75 indicated excellent reliability [14]. An independent-sample t-test was used to compare age, tumor volume, EI, and ADC values between the two groups. The chi-square test was used to compare gender, tumor shape, peritumoral space, cystic necrosis, intratumoral hemorrhage, intratumoral calcifications, flowing void effect, attachment mode to the meninges, dural tail signs, and adjacent bone changes between the two groups. Moreover, the rank-sum test was used to compare T1WI signal intensity, T2WI signal intensity, and the degree of enhancement between the two groups. Logistic regression analyses were used to identify independent factors. Receiver operating characteristic (ROC) curves were used to assess the diagnostic efficacy of each parameter or model in discriminating SFT (WHO grade II) or AM, and the Delong test was used to determine whether the area under the curve (AUC) of each ROC was significantly different. A p value of <0.05 was considered statistically significant. A nomogram was developed based on the outcomes of logistic regression to predict the two tumors. A nomogram is constructed using logistic regression analysis, where significant predictive variables are assigned individual scores based on their contribution to distinguishing the two tumors. Each variable’s score is plotted on a point scale, and the total score is summed to estimate the probability of a specific tumor type. The final prediction is derived by mapping the total score to a probability scale, providing a visual and quantitative tool for clinical decision-making.

## Results

**Figure 2:**
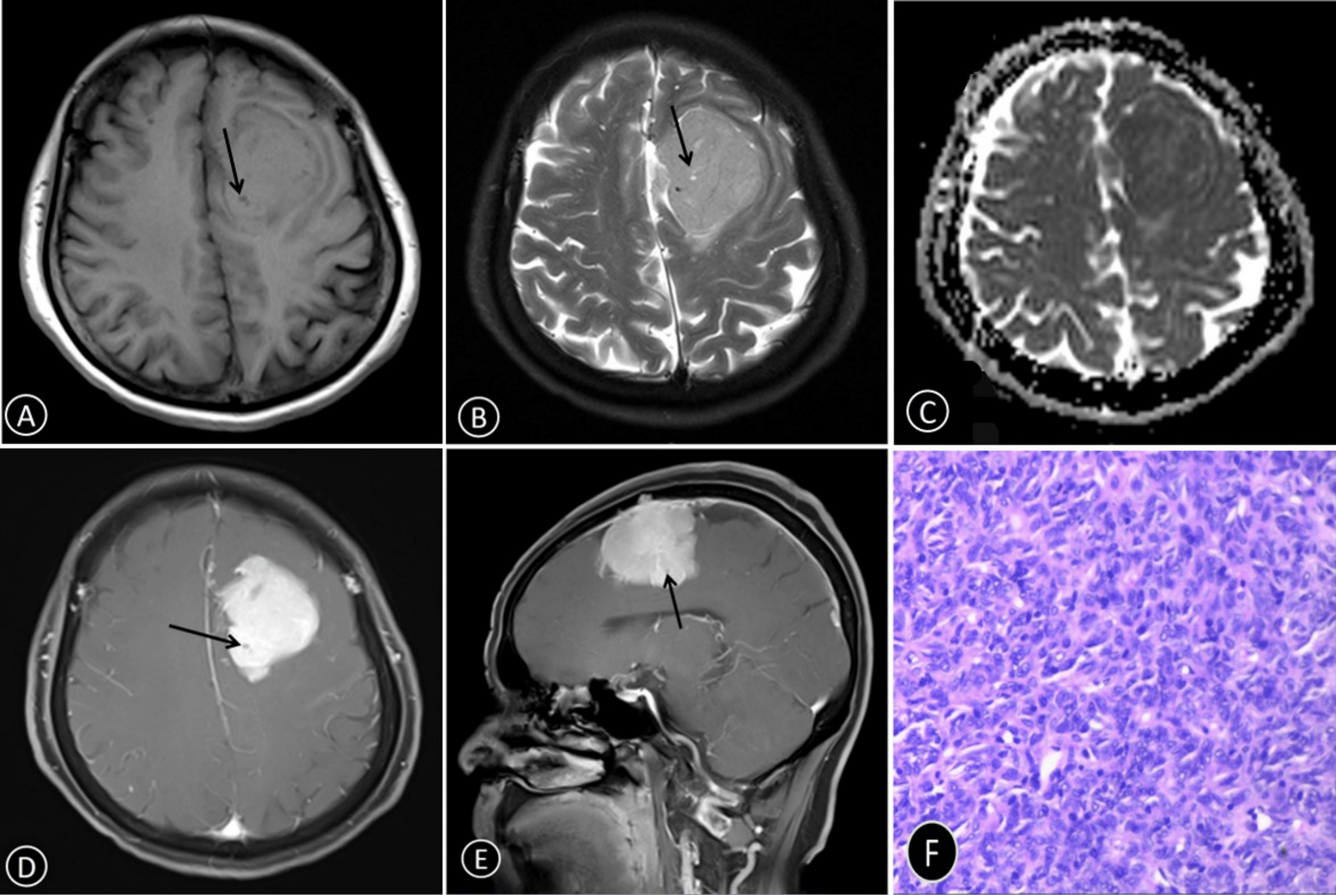
A 67-year-old woman with SFT (WHO grade II) in the left frontal lobe (A) T1WI showing a large mass with isointensity. (B) T2WI showing hyperintensity signal. (C) ADC value was 0.87 × 10^-3^ mm^2^/second. (D,E) Contrast-enhanced T1WI showing heterogeneous moderate enhancement. Cystic necrosis is indicated by the black arrow. (F) H＆E-stained sections (×100) of the tumor core showing that there are many dense, aggregated cells SFT: solitary fibrous tumor; WHO: World Health Organization; T1WI: T1-weighted imaging; T2WI: T2-weighted imaging; ADC: apparent diffusion coefficient; H&E: hematoxylin and eosin

**Figure 3:**
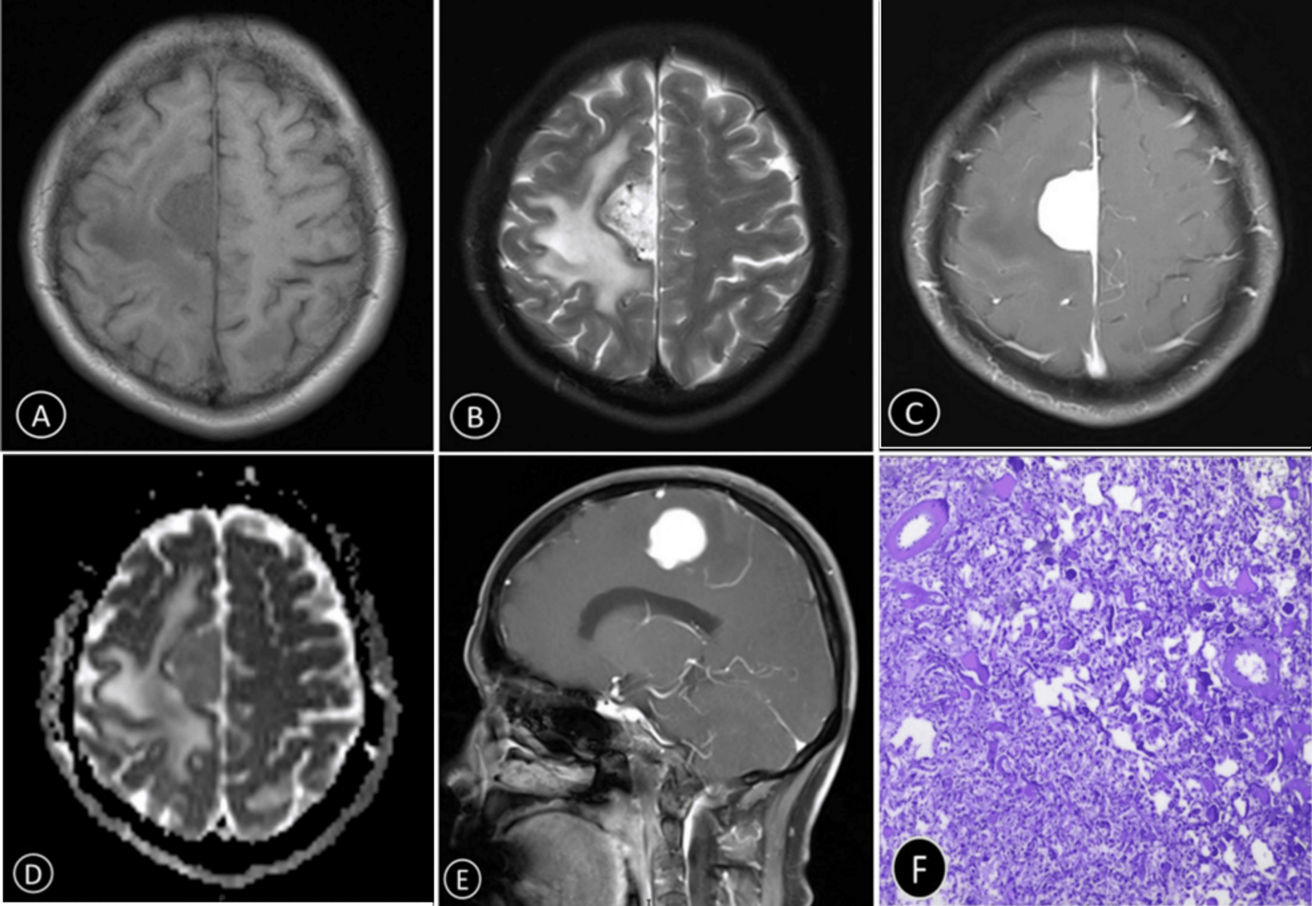
A 60-year-old woman with AM (WHO grade I) in the right frontal lobe (A) T1WI shows a large mass with isointensity. (B) T2WI shows a hyperintensity signal. (C) The ADC value was 1.13 × 10^-3 ^mm^2^/second. (D,E) Contrast-enhanced T1WI shows homogeneous severe enhancement. (F) H＆E-stained sections (×100) of the tumor core showed that there are abundant vascular tissues with varying wall thickness AM: angiomatous meningioma; WHO: World Health Organization; T1WI: T1-weighted imaging; T2WI: T2-weighted imaging; ADC: apparent diffusion coefficient; H&E: hematoxylin and eosin

The interobserver agreement for parameter measurements was excellent (intraclass correlation coefficient, ICC = 0.925), ensuring reliability in assessment. SFT (WHO grade II) tumors were significantly more likely to exhibit a lobulated shape (p = 0.007) and intratumoral cystic necrosis (p = 0.01) compared to AM. In contrast, adjacent bone changes showed no significant difference between the two groups (p = 0.179). Figure 2 represents a case of SFT (WHO grade II), while Figure 3 depicts a case of AM. The parameter measurement results between two observers have good consistency, with an ICC value of 0.925. SFT (WHO grade II) tumors were more likely to exhibit a lobulated shape (95% CI = 0.116-0.648; p = 0.007) and intratumoral cystic necrosis (95% CI = 0.131-0.685; p = 0.01) compared to AM. No significant difference was observed between the SFT (WHO grade II) and AM groups in terms of adjacent bone changes (p = 0.179). However, the SFT (WHO grade II) group exhibited a higher incidence of skull erosion and destruction, whereas AM was more prone to bone hyperplasia and thickening. Table 1 provides further details regarding age, gender tendency, tumor volume, EI, peritumoral space, intratumoral hemorrhage, intratumoral calcifications, flowing void effect, attachment mode to the meninges, and dural tail signs, showing no significant differences between the SFT (WHO grade II) and AM groups (p > 0.05, respectively).

Regarding the T1WI and T2WI signal intensities, the SFT (WHO grade II) group demonstrated significantly higher values than the AM group (95% CI = 0.043-0.883; p = 0.043; 95% CI = -0.977 to -0.082; p = 0.038, respectively) (Table 1). Moreover, the average ADC value of the SFT (WHO grade II) group was notably lower than that of the AM group (95% CI = -0.161 to -0.071; p < 0.001) (Table 2).

**Table 2:** Clinical and MRI characteristics of patients (n = 51) SFT: solitary fibrous tumor; WHO: World health Organization; AM: angiomatous meningioma; EI: edema index; ADC: apparent diffusion coefficient; T1WI: T1-weighted imaging; T2WI: T2-weighted imaging; MRI: magnetic resonance imaging

Variable	Value, n (%)		p value
	SFT (WHO grade II)	AM	
Gender			
Female	10 (43.5%)	16 (57.1%)	0.331^a^
Male	13 (56.5%)	12 (42.9%)	
Age (years)	44.7 ± 15.6	48.3 ± 12.6	0.362^b^
Tumor volume (mm^3^)	56.1 ± 13.6	51.2 ± 10.3	0.150^b^
EI	1.84 ± 1.02	1.77 ± 0.79	0.789^b^
ADC (10^-3 ^mm^2^/second)	1.01 ± 0.09	1.12 ± 0.07	<0.001^b^
Shape			
Lobulated	17 (73.9%)	10 (35.7%)	0.007^a^
Circular	6 (26.1%)	18 (64.3%)	
Peritumoral space			
Yes	19 (82.6%)	22 (78.6%)	0.718^a^
No	4 (17.4%)	6 (21.4%)	
Cystic necrosis			
Yes	14 (60.9%)	7 (25%)	0.010^a^
No	9 (39.1%)	21 (75%)	
Intratumoral hemorrhage			
Yes	1 (4.3%)	2 (7.1%)	0.673
No	22 (95.7%)	26 (92.9%)	
Intratumoral calcifications			
Yes	3 (13%)	4 (14.3%)	0.898^a^
No	20 (87%)	24 (85.7%)	
Flowing void effect			
Yes	11 (33.3%)	15 (53.6%)	0.683^a^
No	12 (66.7%)	13 (46.4%)	
Attachment mode to the meninges			
Wide base	7 (30.4%)	16 (57.1%)	0.056^a^
Narrow base	16 (69.6%)	12 (42.9%)	
Dural tail sign			
Yes	8 (34.8%)	14 (50%)	0.275^a^
No	15 (65.2%)	14 (50%)	
Adjacent bone changes			
Damage	6 (26.1%)	5 (17.9%)	0.179^c^
Proliferation	3 (13%)	10 (35.7%)	
No	14 (60.9%)	13 (46.4%)	
T1WI signal			
1	1 (4.3%)	3 (16.7%)	0.043^c^
2	9 (39.1%)	6 (33.3%)	
3	10 (43.5%)	9 (50%)	
4	3 (13%)	0 (0%)	
5	0 (0%)	0 (0%)	
T2WI signal			
1	0 (0%)	0 (0%)	0.038^c^
2	2 (9.1%)	0 (0%)	
3	10 (45.4%)	11 (39.3%)	
4	9 (40.9%)	7 (25%)	
5	1 (4.5%)	10 (35.7%)	
Enhancement			
Severe	11 (47.8%)	19 (70.4%)	0.152^c^
Moderate	12 (36.4%)	8 (29.6%)	
Mild	0 (0%)	0 (0%)	

**Figure 4:**
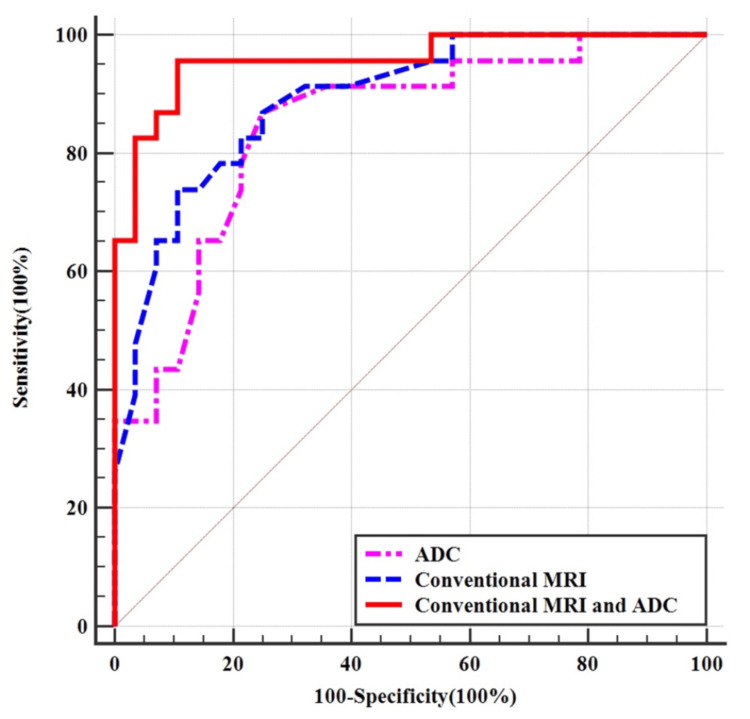
ROC analysis of conventional MRI feature, ADC value and combined conventional MRI feature, and ADC value in differential diagnosis SFT (WHO grade II) and AM ADC: apparent diffusion coefficient; MRI: magnetic resonance imaging; ROC: receiver operating characteristic; SFT: solitary fibrous tumor; AM: angiomatous meningioma; WHO: World Health Organization

When the ADC value fell below 1.08 × 10^-3 ^mm^2^/second, the lesion was diagnosed as SFT (WHO grade II), yielding a sensitivity of 86.9%, a specificity of 75%, and an accuracy of 84.7%. Logistic regression analysis indicated that T1WI and T2WI signal intensities, tumor shape, cystic necrosis, and ADC value were independent factors in the differential diagnosis of AM and SFT (WHO grade II), with ADC value exhibiting the highest diagnostic efficiency. Combining T1WI signal intensity, T2WI signal intensity, tumor shape, cystic necrosis, and ADC value resulted in the highest AUC value of 0.958 (95% CI = 0.861-0.994), with a sensitivity of 95.7%, a specificity of 89.3%, and an accuracy of 92.2%. These values surpassed the diagnostic performance of conventional MRI (95% CI = 0.771-0.961; AUC = 0.891; p = 0.040) or DWI sequence (95% CI = 0.719-0.932; AUC = 0.846; p = 0.015) alone (Figure 4).

**Figure 5:**
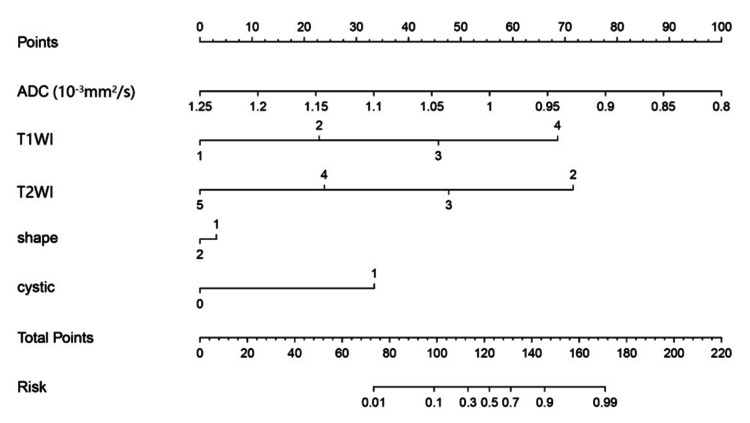
The nomogram for predicting the SFT (WHO grade II) and AM. The value assigned to each factor was scored on a scale of 0-100. By adding scores for each factor, one can obtain a total score. Based on the total score, the probability of two tumors is displayed by projecting the score to the bottom risk axis SFT: solitary fibrous tumor; WHO: World Health Organization; AM: angiomatous meningioma; ADC: apparent diffusion coefficient; T1WI: T1-weighted imaging; T2WI: T2-weighted imaging

**Figure 6:**
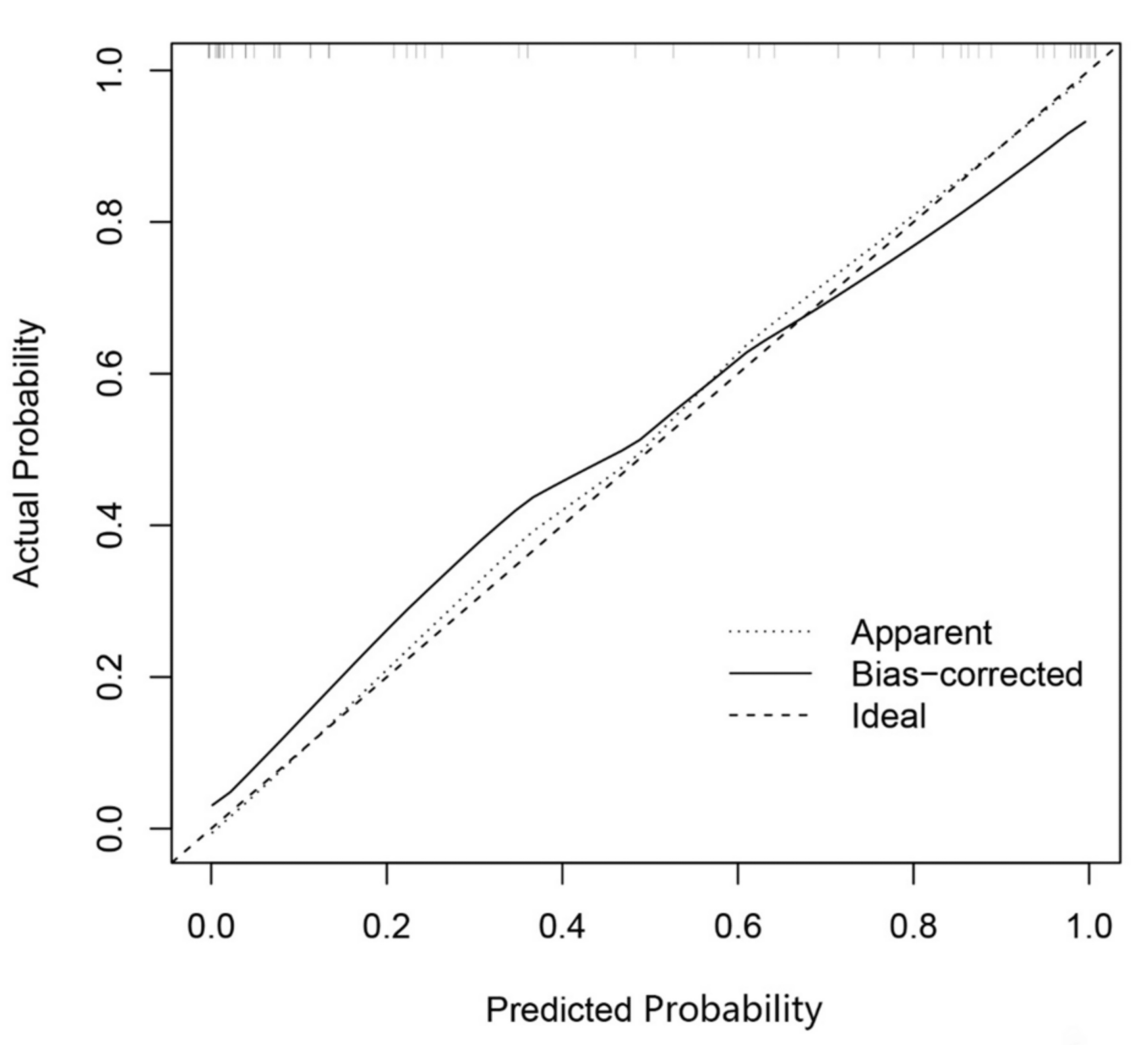
The X-axis is the nomogram-predicted probability of two tumors. The Y-axis is the actual probability. The dotted line represents an ideal standard curve; the solid line represents the prediction calibration curve of the nomogram. The solid line has a closer fit to the ideal dotted line, which indicates better predictive accuracy of the nomogram

The combined model showed that these five independent factors were associated with two tumors and were used to develop a nomogram (Figure 5). As shown in the calibration curve, a good consistency was observed between nomogram prediction values and actual findings (Figure 6).

## Discussion

Previous studies demonstrated the presence of gender in SFT (WHO grade II) and AM was not consistent [15,16]. In this study, unlike the previous one, the gender tendency was found to be insignificant between the two groups. The average age of patients with SFT (WHO grade II) is younger than that of patients with AM, as reported in a previous study [17]. In this study, the age of people with SFT (WHO grade II) is not significantly different from those with AM. This may be due to the small sample size, so increasing the number of patients in further analyses is necessary. Another study reported [18] that the time interval between the onset of clinical symptoms and the diagnosis of SFT (WHO grade II) is shorter than that of the meningioma group. The patients in the SFT (WHO grade II) group were younger than those in the AM group, which may be due to the high degree of malignancy and the rapid growth of the SFT (WHO grade II), which more easily causes clinical symptoms at an early stage.

Conventional MRI is extremely valuable in the diagnosis and differential diagnosis of neoplastic diseases of the CNS [19]. Consistent with previous reports, SFT (WHO grade II) was more likely to exhibit a lobular appearance and a higher likelihood of cystic necrosis than AM, which may be due to the invasive growth of SFT (WHO grade II), different growth rates of tumors in different directions, and the relative lack of need for blood supply caused by the rapid growth of the tumor [7]. In this study, despite adjacent bone changes, there was no significant difference between SFT (WHO grade II) and AM, but the bone in the SFT (WHO grade II) group was more prone to tumor-induced damage, while in the AM group, the bone changes were mainly hyperplasia and thickening, which is similar to the conclusions of Wei et al. [20]. One previous research stated that a malignant SFT (WHO grade II) tumor could easily penetrate the skull. However, being a benign tumor, meningioma often showed cranial compression. The findings of Xiao et al. [19] and He et al. [17] regarding peritumoral edema in SFT (WHO grade II) and AM tumor groups were controversial. He et al. found that peritumoral edema was significantly less in the SFT group than in the AM group. He et al. also suggested that the apparent edema in the AM may be related to vascular endothelial growth factor A and the length of tumor capillaries in it [17]. However, Xiao et al. [19] found no significant difference in peritumoral edema between the SFT (WHO grade II) and AM groups. This finding is consistent with his study. The author considered both SFT (WHO grade II) and AM to be extracerebral tumors. The primary cause of peritumoral edema is vasogenic edema resulting from tumor compression. Moreover, the volume of SFT (WHO grade II) and AM did not significantly differ, indicating that there was no significant difference between the two tumors. There was no significant difference between the two groups regarding the dural tail sign. As noted in a previous study [7], the sign predominantly appears in meningiomas; however, it can also be present in dural masses such as SFT (WHO grade II). However, Fan et al. [21] did not approve the result. According to previous reports [20,22], this study also found no significant difference in intratumoral hemorrhages, intratumoral calcifications, peritumoral space, empty blood vessel flow signal, and mode of attachment to the meninges between SFT (WHO grade II) and AM.

There is still no consensus on whether conventional MRI signals can differentiate between SFT (WHO grade II) and AM, even with the introduced radiomic or deep learning model. In this study, the T1WI signal intensity in SFT (WHO grade II) was higher than that of the AM group, but the T2WI signal intensity was lower than that of the AM group. This conclusion is consistent with the study by He et al. [22], the deep learning model by Chen et al. [15], and the texture model by Dong et al. [23]. Perhaps this was due to a pathological feature.

The unified exponential DWI model has become a widely used clinical MR functional sequence due to its short scan time, ability to detect movement of water molecules, and better reflection of tumor cell density. Consistent with the study results by Shankar et al. [9], this study also showed that the mean ADC in the SFT (WHO grade II) group was significantly lower than in the AM group, which may be due to the histopathology of the two tumor groups. Microscopically, in SFT (WHO grade II) tumors, the tumor cells mainly consisted of dense spindle-shaped cells, and blood vessels of various sizes could be seen. In contrast, in AM, meningeal epithelial cells were scattered among hyperplastic blood vessels. According to He et al. [22], it is more efficient to analyze the difference in ADC values between hemangioma meningiomas and SFT (WHO grade II) tumors using a histogram, which makes it possible to increase the sensitivity and specificity when distinguishing these tumors. However, Liu et al. [24] did not consider the average value of ADC to be useful as a parameter for distinguishing between SFT (WHO grade II) and AM. This contradiction may be related to the algorithm to determine the value of the ADC using a single exponential model. The ADC value theoretically reflects the density of tumor cells in the detected area. Because SFT (WHO grade II) and AM are rich in microvessels, the ADC value is affected by vascular microperfusion. Although the significance of DWI, in combination with the mean ADC of the tumor parenchyma in differentiating hepatic fibrosis (HF) from AM, needs further discussion, DWI, as a widely used MRI method in the clinic, can be used as an important adjunct to conventional MRI examination in differentiating HF. The ROC curve shows that when the mean ADC value of the parenchymal part of the tumor is less than 1.08 × 10^-3^ mm^2^/second, the tumor is usually diagnosed as SFT (WHO grade II), and the accuracy is 82.3%. Meanwhile, the combination of T1WI, T2WI signal, tumor shape, cystic necrosis, and ADC value in the differential diagnosis of AM and SFT (WHO grade II) had the highest diagnostic efficiency and an accuracy of 90.2%, which was higher than the diagnostic value of conventional MRI or DWI sequence. Thus, the combined conventional MRI and DWI sequence could better distinguish SFT (WHO grade II) from AM.

This study still has limitations: 1) a further increase in the number of reported cases and more accurate results is needed and 2) due to the pathological characteristics of the two groups, the ADC parameter was inevitably affected by microperfusion, leading to biased results. Thus, intravoxel incoherent motion imaging will be introduced in the future to study the differences between the two groups of tumors, which uses a double exponential model to separate diffusion from perfusion.

In conclusion, preoperative conventional MRI and DWI sequences offer valuable information for accurate differential diagnosis. SFT (WHO grade II) is more prevalent in patients with a lobulated appearance, heterogeneous MRI signal, cystic necrosis, and significantly lower ADC values. These findings underscore the significance of utilizing a combined model to distinguish SFT (WHO grade II) from AM.

## Conclusions

This study identified tumor shape, cystic necrosis, T1WI, T2WI, and ADC values as significant distinguishing factors. Among these, ADC values demonstrated the highest diagnostic performance, with an optimal cutoff of 1.08 × 10⁻³ mm²/second. Logistic regression confirmed these features as independent predictors, and ROC analysis showed that combining ADC with conventional MRI features significantly improved diagnostic accuracy. Furthermore, the nomogram model exhibited strong agreement between predicted and actual outcomes, reinforcing its clinical applicability. This integrated imaging approach provides a robust and practical tool for accurate tumor differentiation, facilitating precise diagnosis, optimized treatment strategies, and improved surgical planning.
